# Upper respiratory tract mycobiome alterations in different kinds of pulmonary disease

**DOI:** 10.3389/fmicb.2023.1117779

**Published:** 2023-03-23

**Authors:** Xingye Xu, Fangping Ding, Xiangqi Hu, Fan Yang, Ting Zhang, Jie Dong, Ying Xue, Tao Liu, Jing Wang, Qi Jin

**Affiliations:** ^1^NHC Key Laboratory of Systems Biology of Pathogens, Institute of Pathogen Biology, Chinese Academy of Medical Sciences and Peking Union Medical College, Beijing, China; ^2^Division of Pulmonary and Critical Care Medicine, Beijing Chaoyang Hospital, Capital Medical University, Beijing, China

**Keywords:** airway mycobiome, interstitial lung disease, bacterial pneumonia, fungal pneumonia, asthma, lung cancer

## Abstract

**Introduction:**

The human respiratory tract is considered to be a polymicrobial niche, and an imbalance in the microorganism composition is normally associated with several respiratory diseases. In addition to the well-studied bacteriome, the existence of fungal species in the respiratory tract has drawn increasing attention and has been suggested to have a significant clinical impact. However, the understanding of the respiratory fungal microbiota (mycobiome) in pulmonary diseases is still insufficient.

**Methods:**

In this study, we investigated the fungal community composition of oropharynx swab (OS) samples from patients with five kinds of pulmonary disease, including interstitial lung disease (ILD), bacterial pneumonia (BP), fungal pneumonia (FP), asthma (AS) and lung cancer (LC), and compared them with healthy controls (HCs), based on high-throughput sequencing of the amplified fungal internal transcribed spacer (ITS) region.

**Results:**

The results showed significant differences in fungal composition and abundance between disease groups and HCs. *Malassezia* was the most significant genus, which was much more abundant in pulmonary diseases than in the control. In addition, many common taxa were shared among different disease groups, but differences in taxa abundance and specific species in distinct disease groups were also observed. Based on linear discriminant analysis effect size (LefSe), each group had its characteristic species. Furthermore, some species showed a significant correlation with the patient clinical characteristics.

**Discussion:**

Our study deepened our understanding of the respiratory tract mycobiome in some diseases that are less studied and identified the commonalities and differences among different kinds of pulmonary disease. These results would provide the solid basis for further investigation of the association between the mycobiome and pathogenicity of pulmonary diseases.

## Introduction

The respiratory tracts of healthy individuals have shown that they are not sterile as formerly thought but are composed of a complex microbial community termed the microbiome (Erb-Downward et al., 2011). The respiratory microbiome has been suggested to play an essential role in the pathogenesis of pulmonary diseases (Nguyen et al., 2015). The contribution of bacteria to the pathogenesis of pulmonary diseases has been extensively studied, whereas the roles of fungal species are much less understood (de Dios Caballero et al., 2022). In the last few years, the airway fungal microbiota (mycobiome) has drawn closer attention for the following reasons. The respiratory tract is the main portal of entry for various microorganisms, including fungal spores. Based on the microorganism characteristics, the human immune response will either clear them or allow them to colonize the respiratory tract (Pashley et al., 2012; Denning et al., 2014). As a continuous host–pathogen interaction environment, fungi in the respiratory tract are considered to be associated with chronic respiratory diseases (CRDs), including cystic fibrosis (*CF*), chronic obstructive pulmonary disease (COPD), asthma, and bronchiectasis (Nguyen et al., 2015). Growing evidence has shown a significant impact of the airway mycobiome on the clinical outcome of these diseases (Chotirmall et al., 2010; Fillaux et al., 2012). In addition to well-known fungal pathogens such as *Aspergillus fumigatus* and *Candida albicans,* a variety of other fungal species have been described to be involved in pulmonary diseases (Marguet et al., 2012). In addition, the widespread use of antifungal reagents facilitates the emergence of respiratory mycoses that are resistant to common antifungal drugs, posing great challenges to therapy strategies (Vermeulen et al., 2013).

The conventional approach to diagnose fungal infection is culture of respiratory samples. This method is useful for isolating and phenotyping microorganisms but has some limitations. A majority of fungal species are uncultivable on common culture media and many are unknown fungal pathogens (Paniz-Mondolfi et al., 2012). The next-generation sequencing (NGS) approach provided an indisputable method for identifying almost all the fungal species in a sample, including those that were undetected by classical culture methods. This approach could provide a comprehensive view of fungal ecology in various environments and enable the investigation of specific microbe markers in the pathogenesis and progression of diseases (McTaggart et al., 2019). Based on NGS technology, the mycobiomes of a number of pulmonary diseases have been investigated. A study of sputum samples from four *CF* patients suggested that the respiratory mycobiome was involved in the development of lung pathology and that *Candida* was the most commonly observed genus in *CF* (Delhaes et al., 2012). In the analysis of both bronchoalveolar lavage fluid (BALF) lower respiratory tract samples and oropharyngeal wash (OW) upper respiratory tract samples, the respiratory mycobiome showed a markedly different pattern between lung transplant patients and healthy subjects, indicating a possible fungal role in the transplant outcome (Charlson et al., 2012). A study comparing induced sputum samples of asthma patients with control subjects suggested significantly different fungal compositions between the two groups with representative species in each group (van Woerden et al., 2013). Despite these advances in mycobiome studies, the fungal microbiota and their roles in many kinds of pulmonary disease are still not well defined.

In this study, we analyzed the mycobiome in oropharynx swab (OS) samples of 65 patients with five kinds of pulmonary disease, including 20 patients with interstitial lung disease (ILD), 14 patients with bacterial pneumonia (BP), 11 patients with fungal pneumonia (FP), 8 patients with asthma (AS) and 12 patients with lung cancer (LC), as well as 10 healthy controls (HCs), based on high-throughput sequencing of the amplified fungal internal transcribed spacer (ITS) region. In addition to comparing the differences in the mycobiome between disease groups and HCs, the similarities and differences in fungal composition among the five disease groups were also investigated. These results deepen our understanding of the fungal community in the upper respiratory tract of different pulmonary diseases, thus facilitating further investigation of the association between the mycobiome and pathogenicity of pulmonary diseases.

## Materials and methods

### Study population and ethics statement

A total of 72 patients (7 samples with read numbers lower than 2000 were excluded after sequencing) with various lung diseases from the Respiratory Department of Beijing Chaoyang Hospital were enlisted in this study. Oropharynx swabs (OSs) were obtained from patients and then immersed in a virus sampling tube containing 3 ml of maintenance medium (Yocon, China). The tubes were vortexed for 30 s and then immediately stored at −80 ° C. These OS samples were collected from April 2018 to June 2019. Patients were classified into different subgroups according to the disease type, including ILD, BP, FP, AS and LC. In addition, OSs from 10 healthy volunteers were obtained as controls. Clinical characteristics and patient demographics are shown in Table 1. This study was approved by the Ethics Committee of Beijing Chaoyang Hospital at Capital Medical University and the Ethics Committee of the Institute of Pathogen Biology, Chinese Academy of Medical Sciences & Beijing Union Medical College. All patients provided written informed consent to participate.

**Table 1:** Demographics and clinical characteristics of the participants.

	HC	ILD	BP	FP	AS	LC	*p*-value
*Characteristic*							
Age, *y* (mean (SD))	45.90 (6.98)	60.05 (6.69)	54.43 (9.72)	55.82 (8.59)	50.88 (10.70)	56.42 (11.56)	0.0045
Male n (%)	6 (60%)	10 (50%)	7 (50%)	5 (45.45%)	4 (50%)	7 (58.33%)	0.98
BMI (mean (SD))	23.26 (3.15)	24.60 (3.76)	25.04 (2.75)	22.59 (4.71)	26.06 (5.27)	24.2 (3.35)	0.39
Smoking status *n* (%)							0.72
Current	3 (10)	2 (20)	3 (14)	2 (11)	3 (8)	4 (12)	
Former	1 (10)	4 (20)	2 (14)	1 (11)	0	3 (12)	
Never	6 (10)	14 (20)	9 (14)	8 (11)	5 (8)	5 (12)	
*Spirometry*							
FEV1% predicted (mean (SD))		83.56 (21.83)	77.16 (19.37)	78.92 (15.87)	59.50 (24.10)	78.74 (18.66)	0.09
FEV1/FVC ratio (mean (SD))		94.15 (14.30)	83.26 (18.17)	78.73 (14.14)	60.80 (8.51)	69.69 (15.82)	<0.0001
*Medication n (%)*							
Antibiotics		11 (55%)	10 (71.43%)	7 (63.64%)	4 (50%)	5 (41.67%)	0.62
Anti-fungal		2 (10%)	0	4 (36.36%)	0	0	0.0081
Glucocorticoid		2 (10%)	1 (7.14%)	1 (9.09%)	4 (50%)	1 (8.33%)	0.036

Data are presented as mean (SD) or *n* (%). Statistical significance was calculated using ANOVA analysis.

### DNA extraction and fungal ITS sequencing

The OS samples were centrifuged at 18,800 g for 15 min to obtain the precipitate. The precipitate was redissolved in lysis buffer containing snailase, chitinase and lyticase at a 5% concentration and incubated at 37 ° C for 30 min. Then, genomic DNA was extracted using the DNeasy Plant Mini Kit (QIAGEN, Valencia, CA, United States) according to the manufacturer’s instructions. For each batch of extractions, a negative control was performed in parallel that contained all reagents and used distilled water instead of an OS sample. The DNA concentration was determined using a Nanodrop. Extracted DNA was stored at −80°C prior to PCR studies.

The fungal ITS2 region was amplified using the primers gITS7ngs and ITS4ngs, and each primer was barcoded to facilitate library construction. The barcoded primer sequences are listed in Supplementary Table S1. PCR was performed using Q5 High-Fidelity DNA Polymerase (New England BioLabs, Ipswich, MA, United States) according to the protocol. The reaction conditions were as follows: 98°C for 5 min; 35 cycles of 98°C for 20 s, 52°C for 20 s, and 72°C for 20 s; and 72°C for 2 min. Amplified products were purified with AMPure XP beads (Beckman Coulter, Beverly, United States), and library quality was assessed with the Bioanalyzer 2100 system (Agilent, Palo Alto, CA, United States). Finally, all the amplicon libraries were paired-end sequenced on the Illumina HiSeq2500 PE250 system.

### Quality control and raw data processing

The sequenced reads were filtered first. The reads that met the following criteria were removed: for either end of the reads, the adaptor is longer than 5 bp; reads contain more than 50% bases with quality score (Q score) less than 20; the percentage of “N” in a read is more than 5%. Then the overlapping paired-end reads were stitched together to obtain raw tags using flash (version 1.2.11), with a minimum required overlap length of 6 bp and a maximum overlap length of 130 bp (Magoc and Salzberg, 2011). The raw tags were filtered using Trimmomatic (version 0.36) to remove adaptors and low-quality reads (Bolger et al., 2014). Chimeric sequences were identified and removed using UCHIME (version 4.2), and the ITS sequences were determined using ITSx (Version 1.1.2) (Edgar et al., 2011). After these processes, the final obtained sequences were considered effective tags.

### Statistical analysis

The sequence taxonomic distribution and operational taxonomic units (OTUs) were clustered using QIIME 1 (version 1.8.0) by blasting against the UNITE database (sh_general_release_s_10.10.2017) with 97% similarity (Caporaso et al., 2010). Samples with read numbers lower than 2000 were excluded from further analysis. The α-diversity was measured using Mothur (v.1.30) based on the Chao1 index (measuring species richness for low abundance datasets), Shannon index (measuring richness and evenness), and observed OTUs (measuring the number of distinct OTUs) (Schloss et al., 2009). Differences between each two groups were analyzed *via* the Student’s *t*-test, and the *p* values were corrected for multiple testing using Benjamini–Hochberg procedure (Ghosh, 2020). The β-diversity was determined based on principal coordinate analysis (PCoA) analyses using vegan (version 2.5–7) and ape (Version 5.4–1) (Paradis et al., 2004). For PCoA analysis, distance matrix was calculated using the altGower index (Anderson et al., 2006). The permutational multivariate analysis of variance (PERMANOVA) analysis was performed to test the significance (Kelly et al., 2015). The receiver operating characteristic (ROC) value was calculated between two groups using GraphPad Prism. The values of certain species for every patients in a group were compared to the values in the other group. The confidence interval was specified as 95%, and *p* < 0.05 was considered significant. The linear discriminant analysis effect size (LefSe) was calculated to identify the character taxa in each group (Segata et al., 2011). The LefSe algorithm used factorial Kruskal-Wallis sum rank test. One-against-all strategy was selected for multi class analysis. Alpha value for the test among classes was set to 0.05. Threshold on the logarithmic LDA score for discriminative feature is set to 2. Correlation analysis of body mass index (BMI) and lung function index to the specific taxa was performed using Spearman’s rho with the ‘corr.test’ function in R package psych v1.8.12.^1^ The significance of the Pearson correlation coefficient was calculated using the *t*-test.

### Nucleotide sequence availability

The sequencing data of the ITS2 amplicon has been deposited in the NCBI Sequence Read Archive database under accession number PRJNA907293.

## Results

### Oropharynx swab samples from participants

OS samples were collected from 82 participants (Supplementary Table S2). Among these, seven samples with read numbers lower than 2000 were excluded from further analysis. The remaining 75 samples were obtained from patients with pulmonary disease that could be classified into five groups, including 20 patients with ILD, 14 with BP, 11 with FP, 8 with AS and 12 with LC, as well as 10 HCs. In addition, two samples of sterile water were used as negative controls (NC-1 and NC-2) during the preparation of sequenced samples. The results showed that these two samples had few sequenced reads of 342 and 285, respectively, suggesting little fungal contamination from reagents and the environment. Demographics and clinical characteristics of the participants are listed in Table 1.

### Overview of the mycobiome in OSs of pulmonary disease patients and HCs

With the amplification of ITS2 sequences, we analyzed the fungal community of all 75 OS samples. Basidiomycota dominated the upper airway at the phylum level, accounting for 63.1% of all the identified phyla, whereas Ascomycota only accounted for 13.7%. Additionally, 11.7% of the identified OTUs belonged to the Plantae kingdom, which might come from the regular exposure to the environment or diet of the participants.

**Figure 1:**
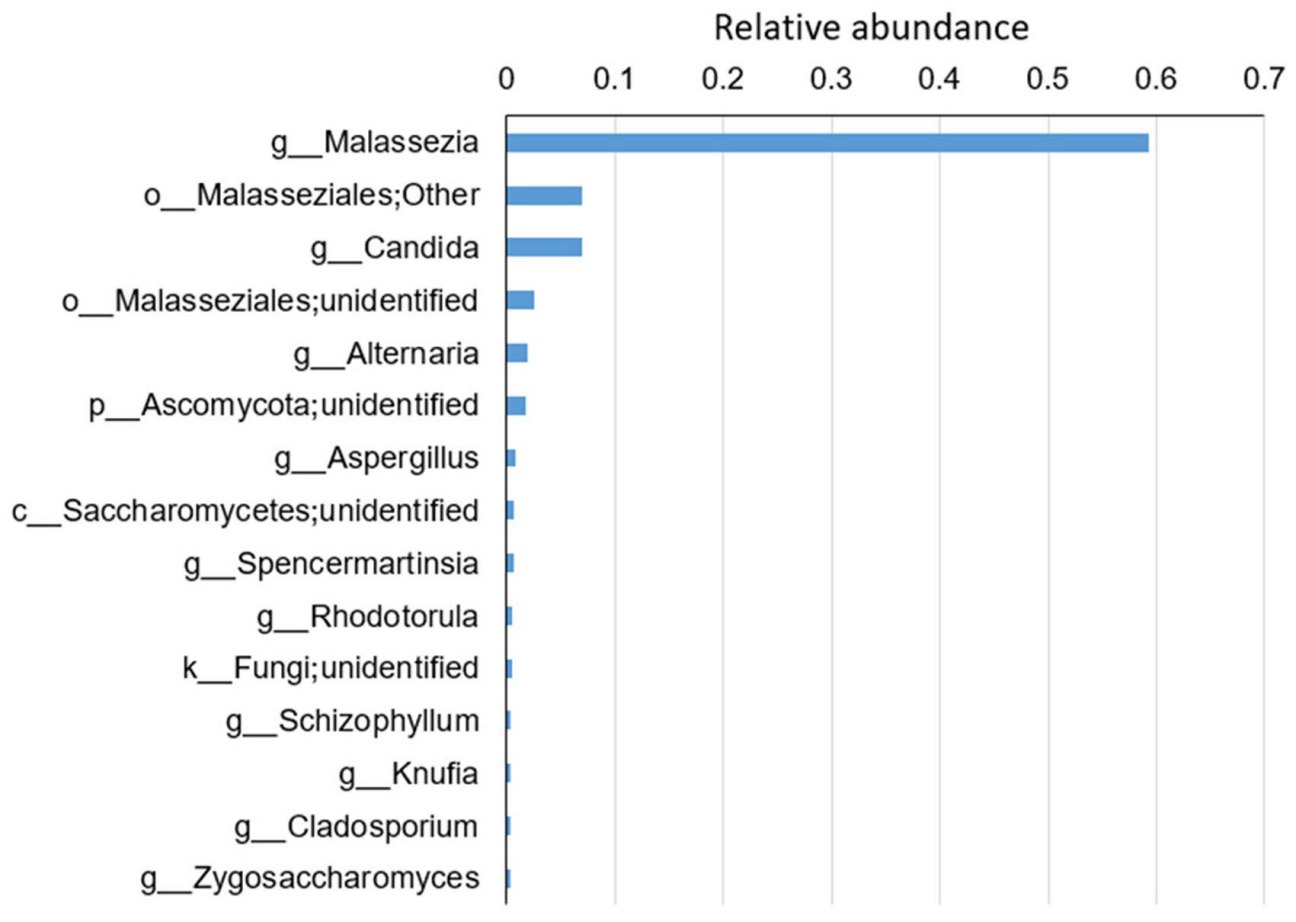
The top 15 most abundant genera on average for all the disease groups and healthy controls. Reads that were not identified at the genus level were grouped at a higher level. “g” indicates genus, “o” indicates order, “c” indicates class and “k” indicates kingdom.

A total of 193 species belonging to 141 genera were identified (Supplementary Table S3). On average, *Malassezia* represented the dominant genus with three identified species, *M. restricta*, *M. globose* and *M. slooffiae*, followed by the *Candida* genus with eight identified species, *C. railenensis*, *C. glabrata*, *C. santamariae*, *C. tropicalis*, *C. tetrigidarum*, *C. albicans*, *C. zeylanoides* and *C. ergatensis*. Other relatively highly abundant genera included *Alternaria*, *Aspergillus*, *Spencermartinsia*, *Rhodotorula*, *Schizophyllum*, *Knufia*, *Cladosporium*, and *Zygosaccharomyces*, comprising the 15 most abundant genera on average of all the sequenced samples (Figure 1). In addition, the top 15 most abundant genera in each group are shown in Supplementary Figure S1.

### Comparison of fungal diversity between disease groups and HCs

**Figure 2:**
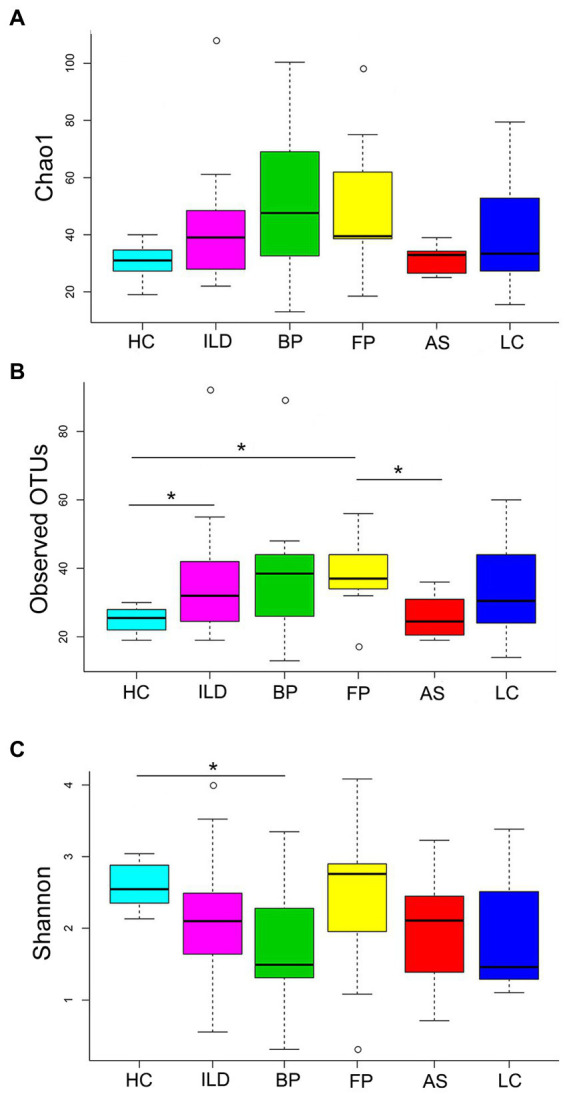
The α-diversity analysis of the identified OTUs. **(A)** Chao1; **(B)** observed OTUs; **(C)** Shannon index. Significant differences between each pair of groups were calculated with Student’s *t*-test, and the *p* values were corrected for multiple testing. * indicates *P*_adj_ < 0.05.

The α-diversity was analyzed based on Chao1, observed OTUs and Shannon index (Figure 2). Chao1 and observed OTUs showed similar results: HC and AS exhibited low richness in comparison to ILD, BP and FP, which were relatively high in richness. However, the Shannon index showed different richness patterns: BP and LC had relatively low values, while FP had the highest Shannon index.

**Figure 3:**
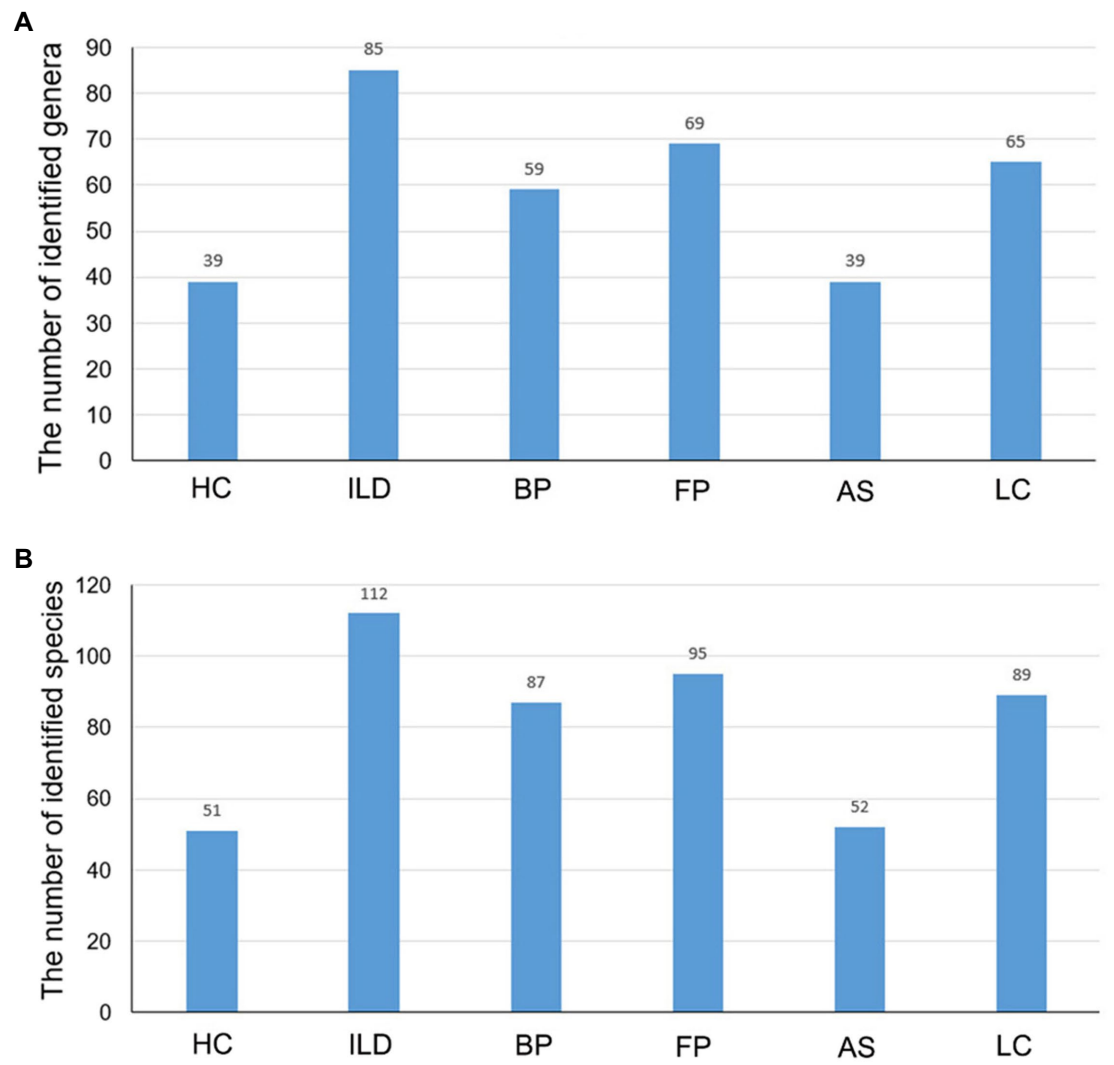
The number of identified taxa for each disease group and healthy control group. The identified **(A)** genera and **(B)** species for each group.

When comparing the number of identified OTUs between different groups, HC and AS had the least. The AS group had 52 identified species belonging to 39 genera, and the HC group had 51 species belonging to 39 genera. The OTUs identified in the other four groups were much more abundant, and ILD had the most identified OTUs, with 112 species belonging to 85 genera, followed by FP, LC and BP (Figure 3).

**Figure 4:**
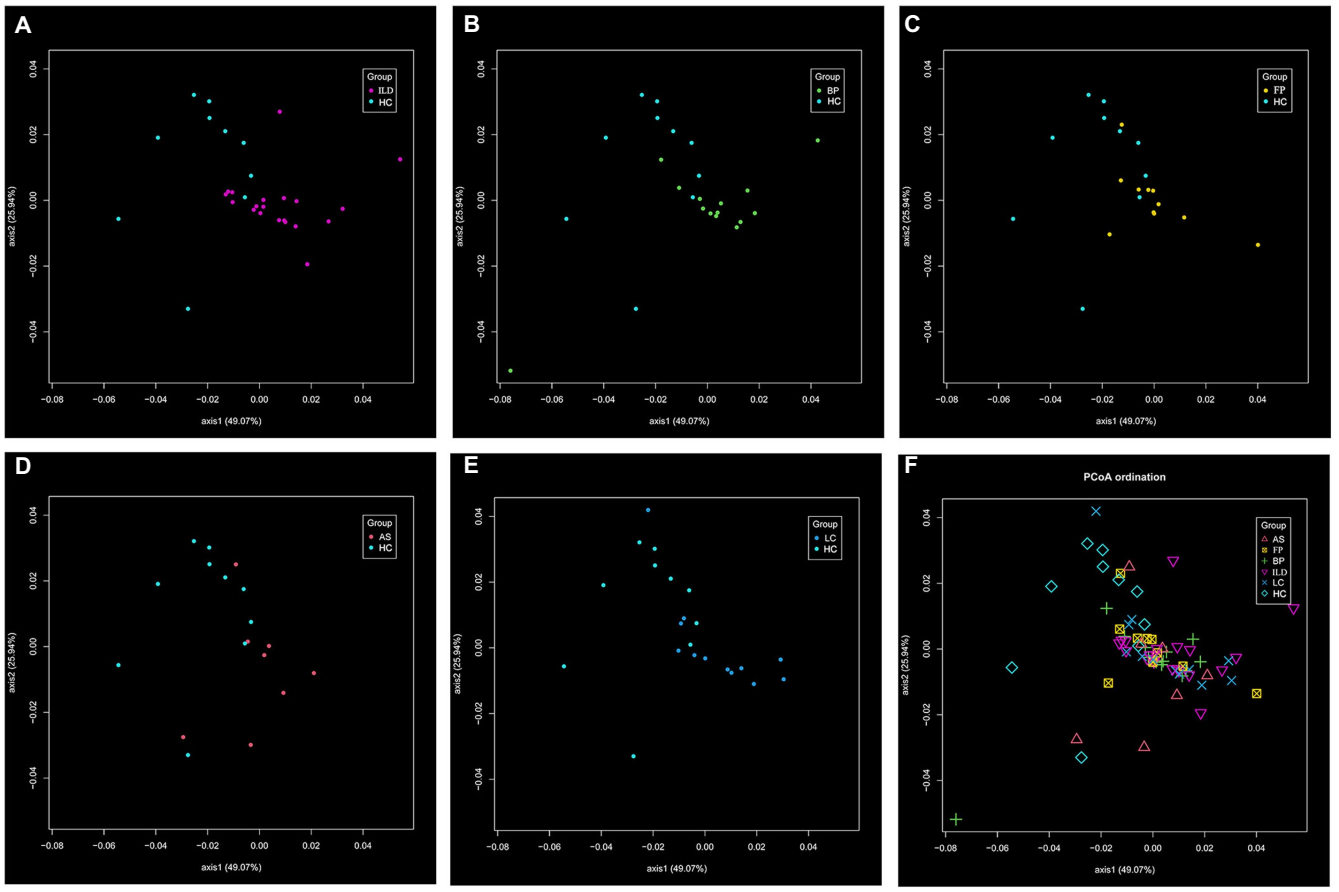
The β-diversity of the identified OTUs. The distance matrix was calculated using the altGower index. The significant difference was calculated with PERMANOVA analysis, and suggested by *P*_adj_ value. The comparison of **(A)** ILD vs. HC; **(B)** BP vs. HC; **(C)** FP vs. HC; **(D)** AS vs. HC; **(E)** LC vs. HC (*P*_adj_ < 0.05); **(F)** among the six groups (significant difference existed between BP and LC, *P*_adj_ < 0.05).

The β-diversity was determined by PCoA. In the comparison of each pair of disease group vs. the HC group as shown in Figures 4A–E, LC group showed significant difference from the HC group. When comparing all 6 groups together, the 5 disease groups showed less difference. Significant difference only existed between BP and LC groups (Figure 4F).

### Different abundances of fungal OTUs between disease groups and HCs

**Figure 5:**
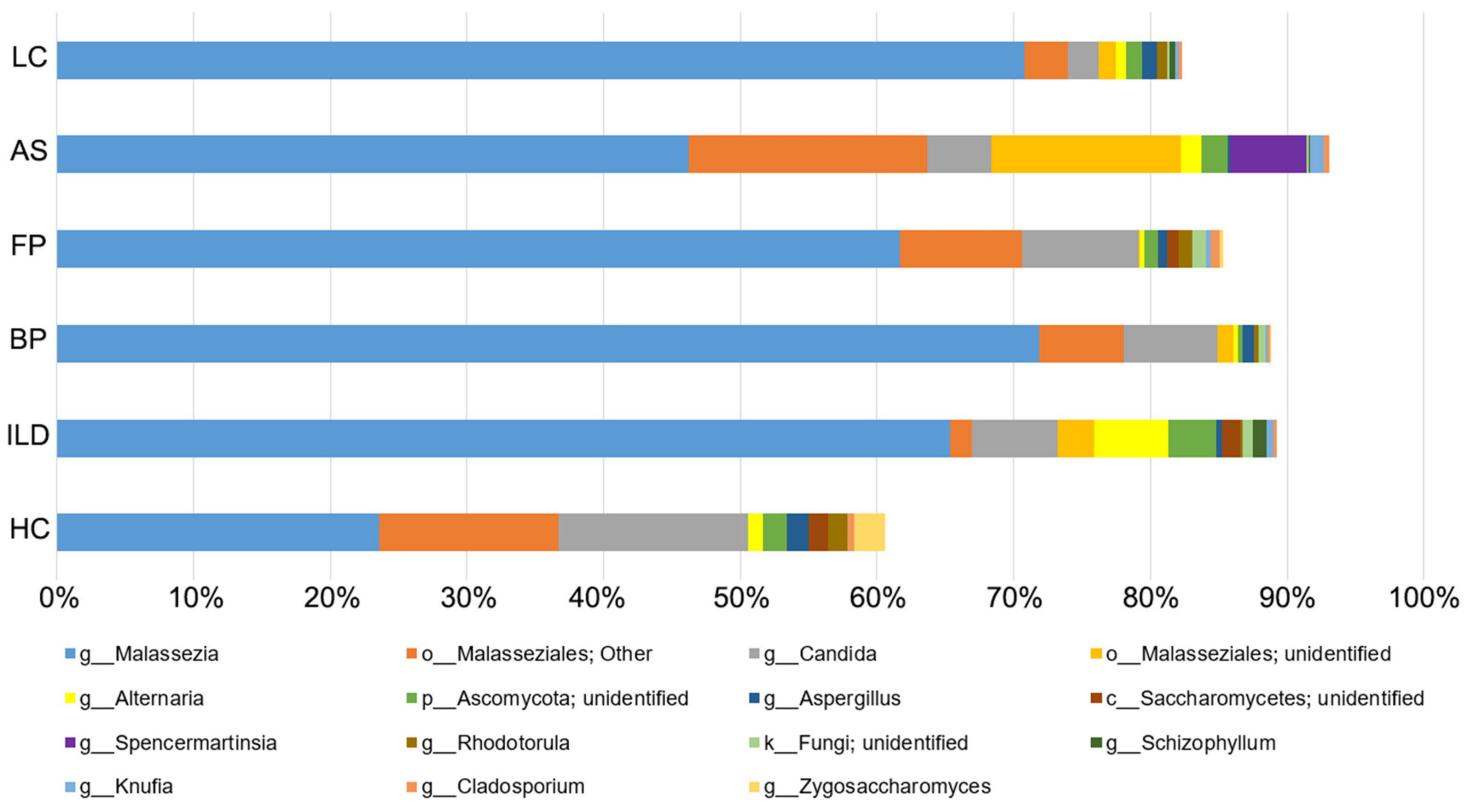
The relative abundance of the identified OTUs at the genus level in each group. The top 15 abundant OTUs on average in all the groups were included.

**Figure 6:**
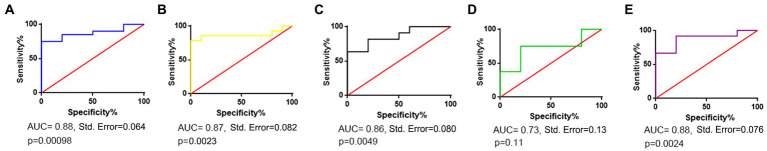
Receiver operating characteristic (ROC) curve corresponding to differences between each disease group and the healthy control group. **(A)** ILD vs. HC; **(B)** BP vs. HC; **(C)** FP vs. HC; **(D)** AS vs. HC; **(E)** LC vs. HC.

The top 15 abundant OTUs on average at the genus level were compared among different groups. As shown in Figure 5, *Malassezia* was the most abundant genus in all five disease groups, accounting for 65.4% in ILD, 71.9% in BP, 61.7% in FP, 46.2% in AS and 70.8% in LC. In contrast, *Malassezia* accounted for only 23.5% in HC. The ROC curve of four groups, including ILD, BP, FP and LC, showed outstanding discriminating quality corresponding to the HC. The AS group did not show a significant difference from the HC group (Figure 6). Two *Malassezia* species, *M. restricta* and *M. globosa,* were among the top three most abundant species. When compared at the species level, *M. restricta* was also much more abundant in ILD (AUC = 0.85, Std. Error = 0.072, *p* = 0.002), BP (AUC = 0.85, Std. Error = 0.082, *p* = 0.004), FP (AUC = 0.79, Std. Error = 0.10, *p* = 0.02) and LC (AUC = 0.92, Std. Error = 0.064, *p* = 0.001) patients than in the HCs. *M. globosa* was significantly more abundant in FP patients (AUC = 0.76, Std. Error = 0.11, *p* = 0.04) than in HCs, further suggesting that *Malassezia* was the most distinct fungus between pulmonary disease patients and HCs (Supplementary Table S4).

*Candida* and *Aspergillus* also had different abundances between disease groups and HCs. *Candida* accounted for 13.9% in HCs but was less abundant in the disease groups, including 6.3% in ILD, 6.9% in BP, 8.5% in FP, 4.6% in AS and 2.2% in LC, although significant differences only existed for the ILD (AUC = 0.74, Std. Error = 0.12, *p* = 0.03) and LC (AUC = 0.77, Std. Error = 0.12, p = 0.03) groups, compared to the HC group. Two *Candida* species, *C. railenensis* and *C. glabrata,* also ranked in the top 15 most abundant OTUs at the species level and were all more abundant in the HC group than in the disease groups; however, no significant difference was observed. *Aspergillus* was a relatively less abundant genus than *Malassezia* and *Candida* identified in our study, similarly showing higher abundance in the HC group than in the disease group. However, except for BP (AUC = 0.75, Std. Error = 0.11, *p* = 0.04) and LC (AUC = 0.83, Std. Error = 0.10, *p* = 0.009) compared to the HC, no significant differences were observed between the other three pairs of disease group vs. the HC group.

### The specific OTUs between disease groups and HCs

When comparing the identified OTUs between each disease group and the HC group, each pair of disease group and the HC group showed some distinct OTUs at both the genus (Supplementary Table S5) and species (Supplementary Table S6) levels. For instance, *Aplosporella* was specific to ILD patients compared to HCs, and *Lectera* was specific to LC patients compared to HCs.

When considering the five disease groups as a whole and compared to the HC group, four genera were specific to the HC group, and 102 genera were specific to the disease groups. At the species level, five species were specific to the HC group, and 142 species were specific to the disease groups.

### Common and differences in OTUs among different disease groups

Among these specific OTUs in disease groups compared to HCs, we found that a large number of them were shared in different disease groups, and most of them had similar relative abundances. These results suggested much overlap of fungal microbiota in different kinds of pulmonary disease.

To investigate the specific marker in each disease group, we further compared these disease group-specific OTUs among the five disease groups at both the genus and species levels (Supplementary Table S7). Based on these analyses, 17 genera and 23 species were specific to ILD patients, 8 genera and 12 species were specific to BP patients, 11 genera and 18 species were specific to FP patients, 6 genera and 7 species were specific to AS patients, and 7 genera and 8 species were specific to LC patients.

In addition to these specific species in each disease group, some species that commonly exist in other groups showed different relative abundances among different disease groups (Supplementary Table S4). *M. restricta* was significantly more abundant in LC than in AS patients (AUC = 0.79, Std. Error = 0.10, *p* = 0.03), with relative abundances of 66.2 and 36.5%, respectively. *M. globosa* was more abundant in BP than in LC patients (AUC = 0.75, Std. Error = 0.10, *p* = 0.03), with relative abundances of 13.0 and 4.6%, respectively. In addition, two *Candida* species were more abundant in FP than AS patients, including *C. railenensis* with a relative abundance of 6% in FP and 0.5% in AS (AUC = 0.81, Std. Error = 0.10, *p* = 0.03) and *C. santamariae* with a relative abundance of 0.6% in FP patients and 0.01% in AS patients (AUC = 0.82, Std. Error = 0.10, *p* = 0.02).

### The character fungal taxa in each group illustrated by LefSe analysis

**Figure 7:**
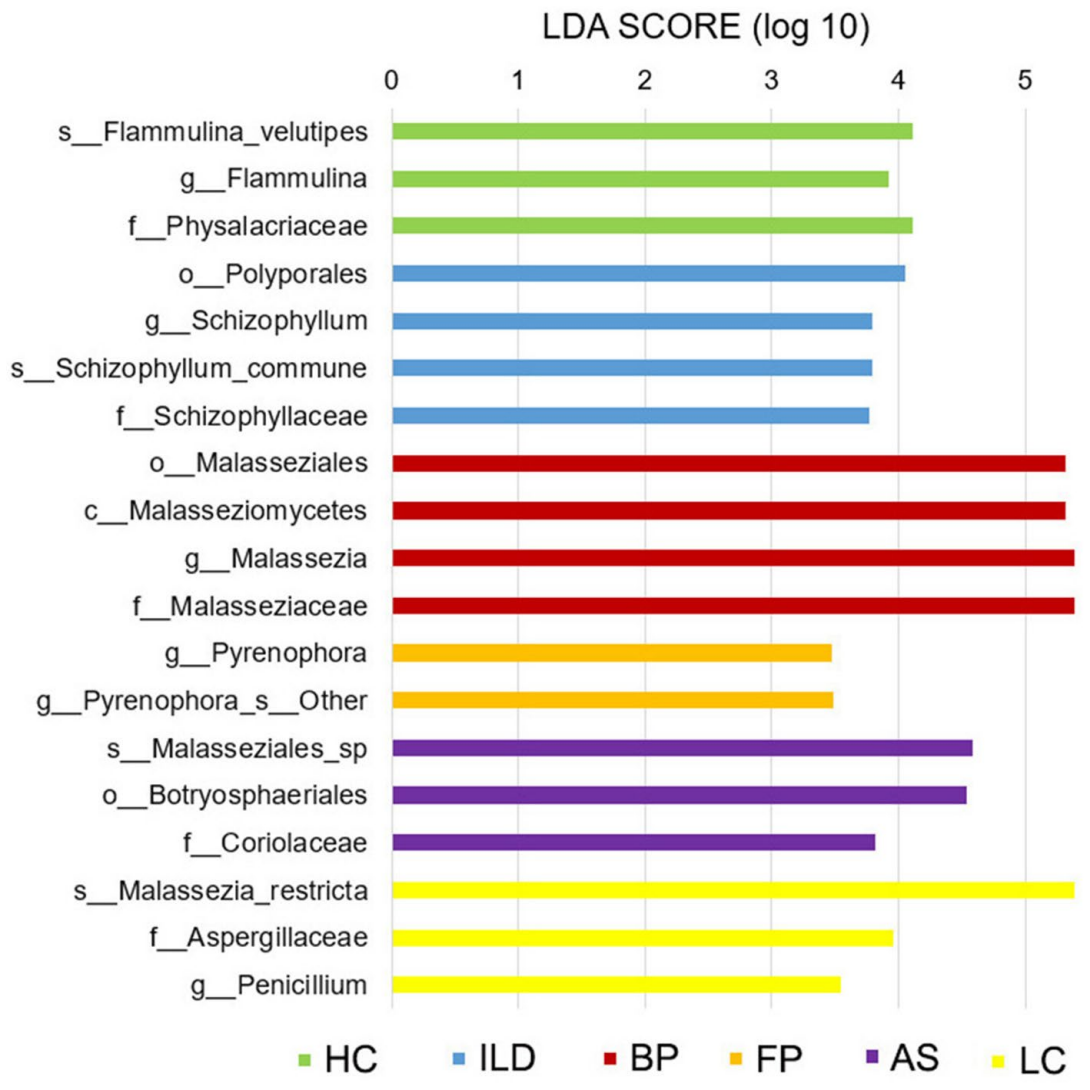
Linear discriminant analysis effect size (LefSe) analysis for all six groups. Taxa shown in each group are considered marker taxa distinguishing from all the other groups with significant difference. LDA scores of >3 are presented (*p* < 0.05).

To detect the character taxa in each group that were significantly different from all the other groups, the LefSe algorithm was performed. As shown in Figure 7, OTUs belonging to the family Physalacriaceae, genus *Flammulina*, and species *F. velutipes* were prominent in HCs. The family Physalacriaceae and the genus *Flammulina* were both specific to the HC group and were not identified in any disease groups. For the five disease groups, each group has its characteristic species. In the ILD group, the genus *Schizophyllum* and the species *S. commune* were significantly abundant. In the BP group, the class Malasseziomycetes was most enriched, and in the FP group, the genus *Pyrenophora* was the most prominent. The AS group was enriched with family Coriolaceae, and the LC group was characterized by the genus *Penicillium* and species *M. restricta*.

### The correlation of mycobiome and patient characteristics

**Figure 8:**
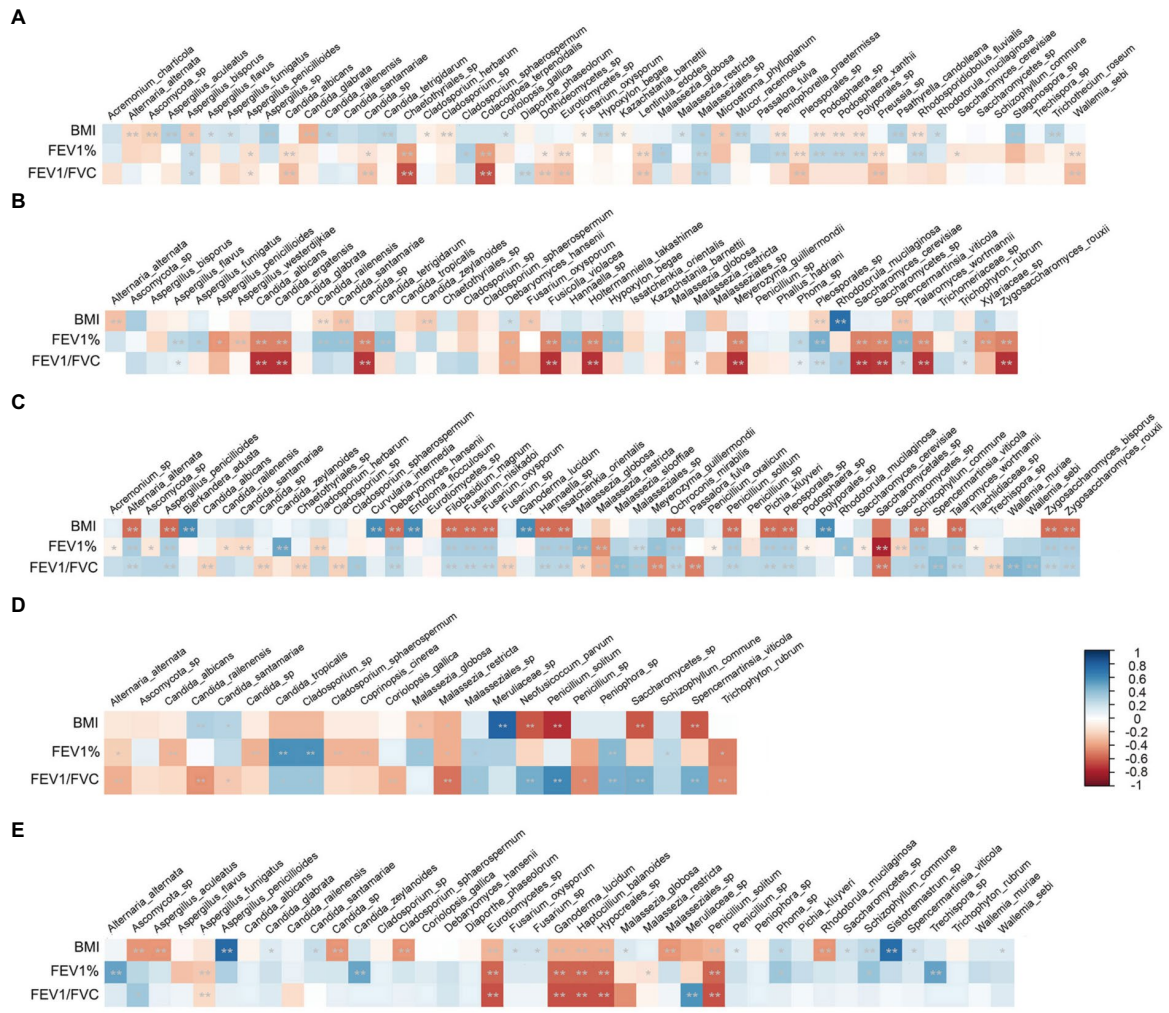
Relations between the patient characteristics and specific species in each disease group. **(A)** ILD group; **(B)** BP group; **(C)** FP group; **(D)** AS group; **(E)** LC group. Stars within heatmap boxes indicate significance (*p < 0.05, **p < 0.01).

To investigate whether the mycobiome composition and taxonomic abundance are associated with patient clinical characteristics, we analyzed the correlation of the mycobiome with BMI; smoking status; lung function index of FEV1% and FEV1/FVC; and therapy approaches, including antibiotic, antifungal and hormone medication, in each disease group separately. The mycobiome did not show any significant relation to smoking status or medication therapy. Instead, BMI and lung function index showed significant correlation to specific species, and these clinically correlated species were distinct in different disease groups. As shown in Figure 8, for instance, BMI was significantly positively correlated with *P. solitum* and negatively correlated with Meruliaceae sp. in the AS group. In the LC group, BMI showed a significantly negative correlation with *A. penicillioides* and *Sistotremastrum* sp.

Lung function also showed a significant correlation with distinct species. Although *Malassezia* was the most significant genus with different abundances between the disease groups and the HC group; the species of this genus did not show a strong correlation with lung function decrease. The dominant species, *M. restricta,* even showed a slight positive correlation with the lung function index in FP and AS patients. Different species of *Candida* showed discrepant relations to lung function as follows: *C. albicans* and other *Candida* sp. in ILD; *C. albicans*, *C. ergatensis* and other *Candida* sp. in BP; *C. albicans*, *C. railenensis*, *C. santamariae* and other *Candida* sp. in FP; and *C. albicans*, *C. railenensis*, *C. santamariae* and other *Candida* sp. in AS were all positively correlated with the lung function index. However, *C. railenensis* and *C. santamariae* in BP, *C. zeylanoides* in FP and LC; and *C. tropicalis* in AS were negatively correlated with the lung function index. In addition to *Candida*, different *Aspergillus* species similarly showed inconsistent correlations with lung function. *A. penicillioides* in ILD; *A. penicillioides* and *A. westerdijkiae* in BP; and *A. fumigatus* in LC were positively correlated with lung function. *A. bisporus* in ILD, *A. flavus* and *A. fumigatus* in BP, and *A. penicillioides* in FP were negatively correlated with the lung function index. In addition, some other species also showed significant and relatively strong negative correlations with lung function, including *P. solitum* in both FP and AS and *Cladosporium* sp. and *Peniophora* sp. in AS.

## Discussion

The human respiratory tract is a complex system that is colonized with various microbes (Zhang et al., 2017). Changes in the diversity and abundance of the airway microbiota are frequently associated with chronic inflammatory pulmonary diseases (Zhang et al., 2017). The bacterial microbiota has been investigated in some of these pulmonary diseases (Fabbrizzi et al., 2019). For instance, in the BALF of patients with ILD, a significantly higher bacterial burden was observed than in the HCs (Molyneaux and Maher, 2013). The microbiome of patients with BP is characterized by low community diversity but increased bacterial burden (Dickson et al., 2014). Although the mycobiome is less diverse and less abundant than the bacteriome, fungal infection-related pulmonary diseases have become increasingly significant clinical problems (Tiew et al., 2020; Ali et al., 2021). Studies have shown that fungi directly contribute to airway inflammation and lung function decline (Amin et al., 2010; Chaudhary et al., 2012; Chotirmall et al., 2013; Liu et al., 2013). Thus, understanding the respiratory tract fungal composition of patients with different kinds of pulmonary disease would have diagnostic and therapeutic significance.

In this study, we investigated the upper respiratory tract mycobiome in patients with five kinds of pulmonary disease and compared it with that in HCs. Based on the analyses of fungi species and abundance, the five disease groups differentiated from the HC group, but among the disease groups, the differences were much smaller. These results suggested that the upper airway tract has different mycobiome compositions between patients with pulmonary diseases and healthy people, and great similarities were shared among the different kinds of pulmonary disease analyzed in our study.

In total, in the five disease groups, Basidiomycota was the most abundant phylum, with an average abundance of 76.55%. In contrast, Basidiomycota only accounted for 40.90% in the HCs. The differences in this phylum between the disease groups and HCs were mainly due to the significant difference in the abundance of the *Malassezia* genus, which was much more abundant in the disease groups than in the HCs.

*Malassezia* is a common part of the human and animal skin mycobiota, which is able to grow at an acidic pH (Soret et al., 2020). Recently, *Malassezia* has been recognized as an emerging infectious pathogen (Delhaes et al., 2012). This genus has been shown to be associated with several chronic inflammatory diseases, including AS, *CF* pulmonary exacerbation (CFPE) and inflammatory bowel disease (IBD) (van Woerden et al., 2013; Sokol et al., 2017; Soret et al., 2020; Bello et al., 2021). In our study, *Malassezia* was identified as the most abundant genus in all five disease groups and was much less abundant in the HC group. The common functions of *Malassezia* in different kinds of pulmonary disease and its specific roles in distinct disease groups need further investigation.

In addition, among the abundant fungal genera identified in our study, many were considered to colonize or act as pathogenic agents in various pulmonary diseases. For example, *Alternaria*, *Cladosporium, Aspergillus*, and *Penicillium* are the predominant aeroallergens that are commonly considered to cause asthma (Hughes et al., 2022). Among these genera, *Alternaria* and *Cladosporium* are unable to grow at body temperature, and their clinical effects are related to exposure levels (Rick et al., 2016). *Furthermore, the s*pore sizes *of Alternaria* and *Cladosporium* are higher than 5 μm*, and they* are mainly deposited in the upper respiratory airways (Frohlich-Nowoisky et al., 2009; Gago et al., 2019). *In our study, Alternaria and Cladosporium* were both identified in all six groups and had similar abundances among the different groups. Thus, we speculated that these two genera identified in the OS samples may be due to daily exposure to the environment. In contrast, *Aspergillus* and *Penicillium are* thermotolerant genera that can colonize the respiratory tract (Rick et al., 2016). Thus, they could be persistent allergenic stimuli and act as opportunistic pathogens (Woolnough et al., 2015). *Aspergillus* is a ubiquitous fungus to which humans are constantly exposed, and *Aspergillus* colonization at the lower respiratory airways is common for patients with *CF*, COPD, AS and even in healthy people (Tong et al., 2018; Gago et al., 2019). Colonization is a prerequisite for subsequent fungal infection, but not all patients colonized with *Aspergillus will* develop disease, which is mainly dependent on the immune status of the host (Gago et al., 2019). *Candida* is also a thermotolerant genus and is frequently found in respiratory tract specimens (Meena and Kumar, 2022). However, the clinical relevance of *Candida* species remains controversial (Erami et al., 2022). In our study, both *Aspergillus* and *Candida* were identified in all six groups. Although no significant differences existed, these two genera were both more abundant in the HC group than in the disease group. The roles of these two commonly observed genera in the respiratory tract and their relations to pulmonary diseases should be studied further. In contrast, *Penicillium* was commonly identified in all five disease groups but was not identified in HCs, suggesting its specific pathogenic role in pulmonary diseases.

One interesting genus identified in our study is *Knufia*, which is a group of extreme stress-tolerant species from the environment, especially rock surfaces (Tesei et al., 2017). *K. epidermidis was first* described in 2008, *and* only several isolates have been reported (Martin-Gomez et al., 2019). *Most* of these isolates were from feet or nails, with little clinical information (Martin-Gomez et al., 2019). *Recently, in 2019, this species was* isolated from a *pediatric dermatological sample* (Martin-Gomez et al., 2019). In our study, *Knufia* was identified in all five disease groups with a relative abundance between 0.1 and 1% but was much less abundant in the HCs with a relative abundance of 0.00087%. Although their natural habitat, source and route of transmission remain unclear, our results provided some potential clues for their role in pulmonary diseases.

In addition to these pathogenic species, the genus *Flammulina*, and one of its species, *F. velutipes,* were only identified in HCs with a relative abundance of 1.6%. *F. velutipes* polysaccharides (FVPs) have been shown to improve gut health by affecting the gut microbiota (Hao et al., 2021). In addition, *F. velutipes* sterols (FVSs) showed potential for the treatment of liver cancer (Yi et al., 2013). However, this genus has not previously been identified in the respiratory tract or digestive tract to the best of our knowledge. This species may also be from a dietary source, and whether it was an accidentally detected species should be explored further.

In addition to the differences in abundance, some species were specific to either the disease group or HC group, further suggesting a different fungal community in the airway tract from pulmonary diseases to the healthy state. When comparing the five disease groups, a large number of taxa were shared by different disease groups, and some species considered potential pathogens or risk factors may be common in different kinds of pulmonary disease. In addition to the common characteristics shared in different pulmonary diseases, some species also showed differences in abundance among distinct disease groups, including *M. restricta, M. globose, C. railenensis* and *C. santamariae.* Furthermore, each disease group had specific species that were not identified in any other groups.

Based on the LefSe algorithm, we identified character species in each group, which were significantly different from all the other groups. Among the above discussed species, the species *F. velutipes* were characterized for HCs; the genus *Malassezia* was significantly abundant in the BP group; and genus *Penicillium* was significantly abundant in the LC group. Another species, *Schizophyllum commune,* which can cause respiratory system infections, was significantly enriched in the ILD group (Itoh et al., 2021). The genus *Pyrenophora* was the character taxa in the FP group. *Pyrenophora* is considered a barley pathogen, and its infection in humans is unclear (Dahanayaka et al., 2021). In our study, this genus was identified in the ILD group at a low abundance and in the FP group at a much higher abundance. Whether it plays a role in respiratory tract diseases should be investigated further.

Furthermore, our observations have potential clinical implications. Some specific species showed significant correlations with patient characteristics in each disease group. *Aspergillus* species were suggested to be positively associated with CFPE and increased inflammatory responses (Gangell et al., 2011; Soret et al., 2020). However, some studies showed inconsistent results in that *Aspergillus* colonization did not show any association with lung function or radiological abnormalities (Bargon et al., 1999; de Vrankrijker et al., 2011). Among the *Aspergillus* species, *A. fumigatus* was the most reported fungal agent colonizing and/or infecting the airways of *CF* patients and is normally considered to have clinical significance (Bouchara et al., 2009; Pihet et al., 2009; Amin et al., 2010; Sudfeld et al., 2010). Some other *Aspergillus* spp., including *A. niger*, *A. flavus*, *A. tubingensis, A. nidulans* and *A. terreus,* were also frequently detected in the airways of pulmonary disease patients (Rougeron et al., 2014; Shahi et al., 2015; Gautier et al., 2016; Rick et al., 2020; Zeng et al., 2021). In our study, *A. fumigatus* showed a slight yet significant negative correlation with the spirometry index only in BP patients but was positively related to spirometry in LC patients. Some other *Aspergillus* species that were negatively correlated with lung function included *A. bisporus* in ILD patients, *A. flavus* in BP patients and *A. penicillioides* in FP patients. *Candida* are normal components of the microflora of the oral cavity, skin, respiratory tract, gastrointestinal mucosa, and genitourinary tract, and are frequently isolated from airway samples in patients with pulmonary diseases, especially in immunocompromised patients (Kumamoto and Vinces, 2005; Meena and Kumar, 2022). Isolation of *Candida* from respiratory specimens is usually considered to indicate colonization, but the clinical relevance of *Candida* colonization remains controversial (Delhaes et al., 2012; Erami et al., 2022). Furthermore, different *Candida* species showed distinct relationships with clinical performance. Colonization of *C. albicans* has been reported to lead to reduced FEV1 values in *CF* patients (Chotirmall et al., 2010; Francis et al., 2021). In another study of adolescent *CF* patients, colonization with *C. glabrata* was shown to be significantly associated with lower lung function, while *C. albicans* colonization was associated with more preserved lung function (Hector et al., 2016). In our study, *Candida* did not show a similar tendency among the five disease groups. *C. albicans* did not correlate with lung function in LC patients, and in the other four disease groups, this species showed a slight positive correlation with the lung function index. Some other *Candida* species, including *C. railenensis* and *C. santamariae* in BP patients, *C. zeylanoides* in FP and LC patients and *C. tropicalis* in AS patients, showed a weak yet significant negative correlation with the lung function index. These inconsistent relations of *Aspergillus* and *Candida* species to lung function may reflect the difference in distinct species and their specific roles in different disease groups. In addition, fungal spp. may not be independent factors of clinical manifestation, and they may also interact with other species. For instance, *C. albicans* was reported to have a synergistic effect with *Pseudomonas* in patients with pneumonia (Roux et al., 2013). Increased virulence of *Pseudomonas*, *E*. *coli and S*. *aureus* was observed in the presence of *Candida* colonization (Meena and Kumar, 2022). Furthermore, *C. albicans* could also promote the biofilm formation of these mixed species, facilitating their antimicrobial resistance and persistence in the respiratory tract (Morales and Hogan, 2010). *A. fumigatus* biofilm formation and conidial germination were also influenced by *P. aeruginosa*, and an antagonistic effect has been proposed between these two species (Mowat et al., 2010).

Some other species also showed significant negative relations to the lung function index. *P. solitum* was significantly negatively related to lung function in both the FP and AS groups. This is consistent with a previous study in which *Penicillium* was suggested to be negatively associated with FEV1 in *CF* patients (Soret et al., 2020). *Cladosporium* sp. and *Peniophora* sp. showed a relatively strong and significant negative correlation with lung function in the AS group. The relations of these species to the lung function decrease deserve further investigation.

Few studies have focused on the fungal composition of airway samples of these five kinds of lung disease. When compared to these limited data, our study validated some of the previous results but also provided some inconsistent conclusions. For instance, a lung microbiota analysis of patients with central lung cancer showed a higher abundance of *Candida* in healthy controls and enrichment of *Malassezia* in patients, which is consistent with our results (Bello et al., 2021). In an analysis of BALF in idiopathic pulmonary fibrosis (IPF) patients, the mycobiome showed no significant difference in the overall mycobiome composition between the IPF group and control group, and both groups were dominated by *Candida* species (Fabbrizzi et al., 2021). In contrast, our study suggested that all the disease groups were dominated by the *Malassezia* genus. To investigate the fungal microbiota and the significant roles of specific species in these pulmonary diseases, many more studies are still needed.

In addition, mycobiome and some particular fungal species have significant roles in the tumourigenesis and the progression of cancer (Gamal et al., 2022). For instance, among subjects with inflammatory bowel disease (IBD), mycobiota dysbiosis is considered to be a trigger factor of colorectal carcinoma (CRC), through chronic inflammation and toxic metabolites secretion, which might cause DNA damage (Vallianou et al., 2021). For the pancreatic ductal adenocarcinoma (PDA) tumor, it showed a 3,000 fold higher abundance of fungi than the normal pancreatic tissue, and the composition of the mycobiome was also distinct in tumor and normal tissue (Aykut et al., 2019). Furthermore, *Malassezia* spp. was markedly enriched in PDA tumors and is a specific species in oncogenic progression, by activating the complement cascade through binding mannose-binding lectin (MBL) to the glycans of the fungal wall. This study suggested that particular fungal species are sufficient to promote the progression of PDA (Aykut et al., 2019). As suggested in our study, *Malassezia* was also the most abundant genus in LC accounting for 70.8% of the mycobiome, which was much more abundant than in the HC that only accounted for 23.5%. In addition, *M. restricta* was identified as a marker species in LC group that showed significant difference from all the other five groups. In accordance with our results, *Malassezia* was significantly enriched in bronchial biopsy samples from patients with central lung cancer compared with HCs (Bello et al., 2021). Few is known about the relations of fungal species and the LC development, however, the roles of *Malassezia* spp. in LC deserve further investigations. The mycobiome could be explored for the diagnosis and prognosis marker as well as therapeutic target of cancers (Narunsky-Haziza et al., 2022).

Limitations of our study exist. First, our study used noninvasive upper airway OS samples to illustrate the airway fungal composition, which is an alternative method for monitoring airway microbiota (Marsh et al., 2016). Previous studies have suggested that although imperfect, upper respiratory tract samples provide a reliable representation of the lung microbiota (Marsh et al., 2016; Al Bataineh et al., 2020). It was hypothesized that pulmonary microorganisms first colonize the oropharynx and then enter the airway tract (Zhang et al., 2020). In spite of this, a further investigation of the fungal microbiota in the low respiratory tract, such as BALF samples, would construct a whole view of the airway mycobiome and would better facilitate identifying fungal biomarkers of different kinds of pulmonary disease. Second, each disease group in our study included a small number of samples, which may not fully represent the features of each kind of disease. A larger cohort of populations should be included to further explore airway mycobiome characteristics and validate the results in this study.

## Data availability statement

The datasets presented in this study can be found in online repositories. The names of the repository/repositories and accession number(s) can be found at: https://www.ncbi.nlm.nih.gov/, PRJNA907293.

## Ethics statement

The studies involving human participants were reviewed and approved by the Ethics Committee of Beijing Chaoyang Hospital in Capital Medical University and the Ethics Committee of the Institute of Pathogen Biology, Chinese Academy of Medical Sciences & Beijing Union Medical College. The patients/participants provided their written informed consent to participate in this study.

## Author contributions

XX and XH performed the experiments and wrote the manuscript. FD collected the samples. FY, TZ, JD, and YX analyzed the data. TL, JW, and QJ designed the research and reviewed the manuscript. All authors read and approved the submitted version.

## Funding

This work was supported by the CAMS Innovation Fund for Medical Sciences (CIFMS) (grant nos. 2021-I2M-1-038 and 2021-I2M-1-039), the National Science and Technology Major Project of China (grant nos. 2018ZX10712001-002-001 and 2018ZX10711001), the Open Project of the State Key Laboratory of Molecular Developmental Biology (grant no. 2022-MDB-KF-14), the National Science and Technology Infrastructure of China (Project No. National Pathogen Resource Center-NPRC-32), and the Non-profit Central Research Institute Fund of Chinese Academy of Medical Sciences (grant no. 2021-PT310-004).

## Conflict of interest

The authors declare that the research was conducted in the absence of any commercial or financial relationships that could be construed as a potential conflict of interest.

## Publisher’s note

All claims expressed in this article are solely those of the authors and do not necessarily represent those of their affiliated organizations, or those of the publisher, the editors and the reviewers. Any product that may be evaluated in this article, or claim that may be made by its manufacturer, is not guaranteed or endorsed by the publisher.

## Supplementary material

The Supplementary material for this article can be found online at: https://www.frontiersin.org/articles/10.3389/fmicb.2023.1117779/full#supplementary-material

## Footnotes

^1^ https://CRAN.R-project.org/package=psych

## Abbreviations

AS: asthma
BALF: bronchoalveolar lavage fluid
BP: bacterial pneumonia
CF: cystic fibrosis
CFPE: cystic fibrosis pulmonary exacerbation
COPD: chronic obstructive pulmonary disease
CRC: colorectal carcinoma
CRD: chronic respiratory disease
FP: fungal pneumonia
HC: healthy control
IBD: inflammatory bowel disease
ILD: interstitial lung disease
IPF: idiopathic pulmonary fibrosis
ITS: internal transcribed spacer
LC: lung cancer
LefSe: linear discriminant analysis effect size
MBL: mannose-binding lectin
NC: negative control
NGS: next-generation sequencing
OS: oropharynx swab
OUT: operational taxonomic units
OW: oropharyngeal wash
PCoA: principal coordinate analysis
PDA: pancreatic ductal adenocarcinoma
ROC: receiver operating characteristic
