# Supplementary material for: Upper respiratory tract mycobiome alterations in different kinds of pulmonary disease
Source: Front Microbiol. 2023 Mar 23;14:1117779. doi: 10.3389/fmicb.2023.1117779 (PMC10076636; doi:10.3389/fmicb.2023.1117779)
Supplement: Supplementary file 1 [file Data_Sheet_1.pdf]

Supplementary Figure

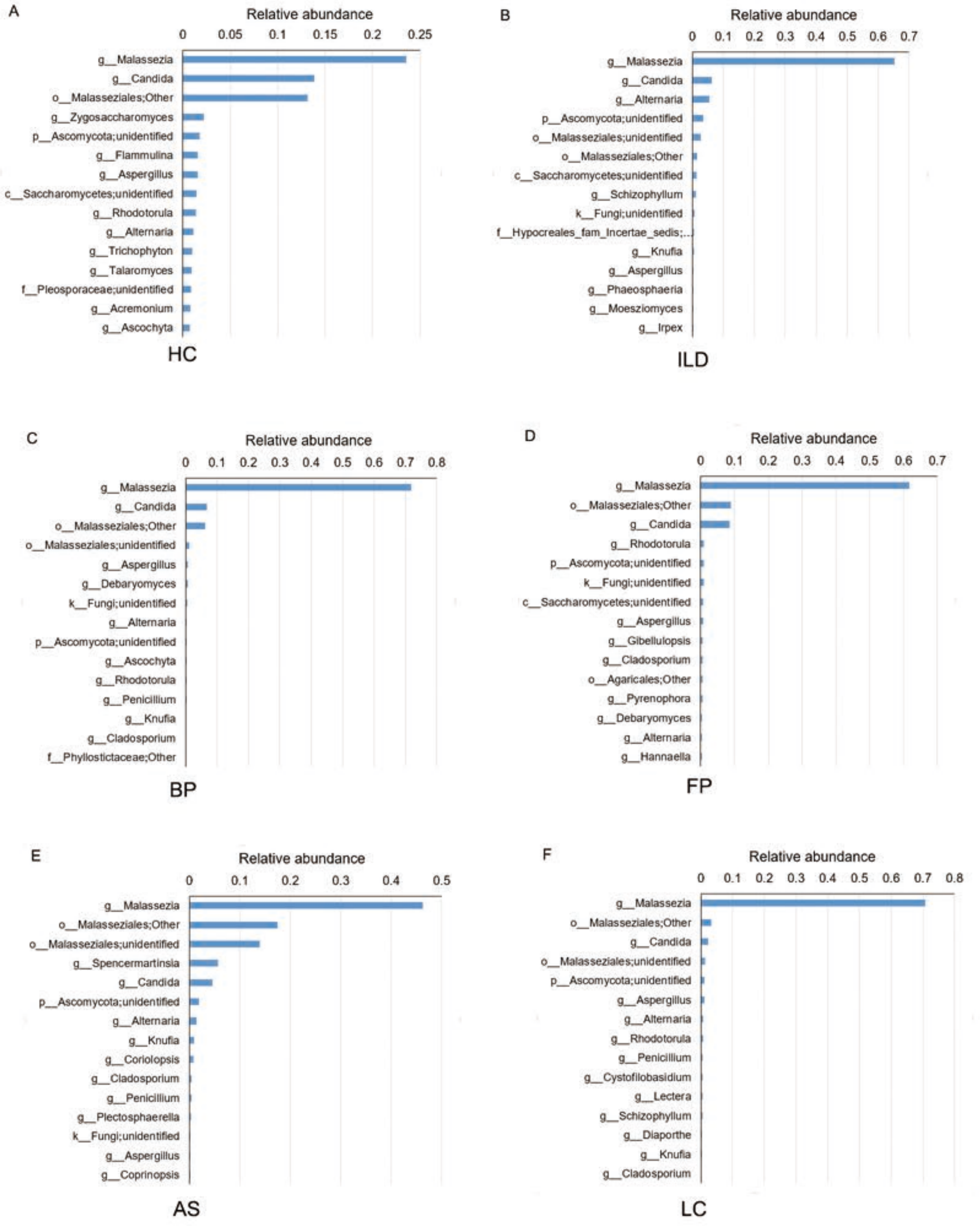

Supplementary Figure S1. The top 15 most abundant genera in each group. (A) The HC group; (B) the ILD group; (C) the BP group; (D) the FP group; (E) the AS group; (F) the LC group. Reads that were not identified at the genus level were grouped at a higher level. "g" indicates genus, "o" indicates order, "c" indicates class and "k" indicates kingdom.
